# Calculating the overall survival probability in patients with cervical cancer: a nomogram and decision curve analysis-based study

**DOI:** 10.1186/s12885-020-07349-4

**Published:** 2020-09-01

**Authors:** Guilan Xie, Ruiqi Wang, Li Shang, Cuifang Qi, Liren Yang, Liyan Huang, Wenfang Yang, Mei Chun Chung

**Affiliations:** 1 Department of Obstetrics and Gynecology, Maternal & Child Health Center, The First Affiliated Hospital of Xi’an Jiaotong University, 277 West Yanta Road, Xi’an, Shaanxi 710061 People’s Republic of China; 2 School of Public Health, Xi’an Jiaotong University Health Science Center, Xi’an, Shaanxi Province People’s Republic of China; 3 Department of Public Health and Community Medicine, Tufts University School of Medicine, Boston, Massachusetts USA

**Keywords:** Cervical cancer, Overall survival, Nomogram, Decision curve analysis

## Abstract

**Background:**

Cervical cancer has long been a common malignance troubling women. However, there are few studies developing nomogram with comprehensive factors for the prognosis of cervical cancer. Hence, we aimed to build a nomogram to calculate the overall survival (OS) probability in patients with cervical cancer.

**Methods:**

Data of 9876 female patients in SEER database and diagnosed as cervical cancer during 2010–2015, was retrospectively analyzed. Univariate and multivariate Cox proportional hazard regression model were applied to select predicted factors and a nomogram was developed to visualize the prediction model. The nomogram was compared with the FIGO stage prediction model. Harrell’s C-index, receiver operating curve, calibration plot and decision curve analysis were used to assess the discrimination, accuracy, calibration and clinical utility of the prediction models.

**Result:**

Eleven independent prognostic variables, including age at diagnosis, race, marital status at diagnosis, grade, histology, tumor size, FIGO stage, primary site surgery, regional lymph node surgery, radiotherapy and chemotherapy, were used to build the nomogram. The C-index of the nomogram was 0.826 (95% CI: 0.818 to 0.834), which was better than that of the FIGO stage prediction model (C-index: 0.785, 95% CI: 0.776 to 0.793). Calibration plot of the nomogram was well fitted in 3-year overall OS prediction, but overfitting in 5-year OS prediction. The net benefit of the nomogram was higher than the FIGO prediction model.

**Conclusion:**

A clinical useful nomogram for calculating the overall survival probability in cervical cancer patients was developed. It performed better than the FIGO stage prediction model and could help clinicians to choose optimal treatments and precisely predict prognosis in clinical care and research.

## Background

Cervical cancer has long been a common malignance troubling women. Although the screening programs for cervical cancer are conducted in many countries and regions, there are still a large number of people dead of advanced stage cervical cancer [1]. It was estimated that there were approximately 311,000 deaths owing to cervical cancer worldwide, which ranked only following the breast cancer [2]. And the proportion of cervical cancer in young women is increasing, which will shorten life expectancy [3]. Tough there are several clinical treatments on cervical cancer, the prognosis of advanced cervical cancer is still poor. The International Federation of Gynecologists and Obstetricians (FIGO) stage system mainly based on clinical examination, is widely used to stage cervical cancer for clinicians to choose specific treatments and predict prognosis [4]. However, the survival of cervical cancer patients differ from each other even with the same clinical stage. Therefore, using FIGO stage system to estimate prognosis of cervical cancer is not entirely satisfactory. And many other factors have been identified related to the survival of cervical cancer [5, 6].

Nomogram is a visualized method of prediction model and it can generate a probability of a clinical event tailored to individual patient [7]. It integrates multiple predictors to provide comprehensively considered probabilities. In recent years, nomogram has gained increasing attention. Some researchers used it in oncology studies, and found that nomogram could precisely predict the oncology diagnosis and prognosis, and perform better than the frequently-used TNM stage system [8, 9]. There were some nomograms built for predicting the survival of cervical cancer, but they tended to be based on small sample size cohorts, which might reduce robustness of the prediction models [10, 11].

Decision curve analysis is a novel method and recommended by several top journals [12–14]. It can calculate net benefit of the prediction models to measure their clinical usefulness, which is significant for the final application of the prediction models. However, in the field of cervical cancer, there are few researches applying it to analyze the net benefit of the prediction models for its newness [10, 11].

In this study, our goal was to develop a clinical useful nomogram to calculate the overall survival probability in cervical cancer patients, based on the sociodemographic characteristics and clinical treatment information. Such a nomogram would be a useful tool helping clinicians to choose optimal treatments in clinical care and research.

## Methods

### Patients

All data was obtained from the Surveillance, Epidemiology, and End Results (SEER) database by SEER*Stat 8.3.6. (https://seer.cancer.gov/seerstat/). SEER database is a database of cancer statistics, collecting information of patients in 18 tumor registries and covering 28% of the total U.S. population [15]. When downloading the SEER*Stat, we all signed and returned the research data agreement to the SEER Program and followed the agreement through the whole study in order to protect the privacy of patients.

A total of 9876 patients were finally included, and the inclusion and exclusion of patients were done through SEER*Stat 8.3.6. by choosing corresponding variables and limitations. Those who were newly diagnosed with cervical cancer during 2010–2015 were included. Those who were less than 18 years old, had multisource tumor and whose information was uncompleted or collected from autopsy or death certificate were excluded. The ending status was dead or censor by November 31st, 2018.

Data included the sociodemographic information (age at diagnosis, race and marital status at diagnosis), pathologic and histologic information (grade, histology, tumor size, FIGO stage, cancer cells in lymph nodes and metastasis), clinical treatments (primary site surgery, regional lymph node surgery, radiotherapy and chemotherapy) and survival information (survival time and ending status). Among them, grade represented pathological grade, including well differentiated (Grade I), moderately differentiated (Grade II) and poorly differentiated (Grade III). FIGO stage was classified by the 2009 FIGO stage system. And the continuous predictors (age and tumor size) were divided into subgroups by previously reported cutoff points [6, 16]. The time calculated from diagnosis to dead or censored was defined as overall survival.

### Statistical analysis

Descriptive statistics was used to embody the baseline characteristics. Kaplan-Meier method was conducted to estimate overall survival rates, and log-rank test was employed to contrast the different subgroups of the variables. Univariate and multivariate Cox proportional hazard regression model were performed to find significant predictors (*P*-value< 0.05). Forward stepwise was conducted by likelihood ratio (LR). A nomogram was built to visualize the prediction model based on the prognostic factors and it was compared to the FIGO stage prediction model which was only based on the FIGO stage.

Bootstrapping 1000 resamples was used to internally validate the predicted ability of the nomogram. Harrell’s C-index was calculated to measure the discrimination of the prediction models, which mirrored their abilities to accurately distinguish patients who were dead and censored. The area under the receiver operating characteristic (ROC) curve (AUC) was used to assess the accuracy of the prediction models in 3-year and 5-year OS prediction.

Calibration plots were drawn to assess the calibration of the nomogram. The predicted probabilities for each cervical cancer patient were listed in orders and divided into ten groups, then compared with the actual probabilities. The calibration plots can measure whether a nomogram is erroneously estimating and overfitting. A prediction model is considered having good calibration when the plot perfectly agrees with the 45-degree line. When the slope is less than 1, it illustrates that the nomogram is overfitting; when the intercept is less than 0, it illustrates that the nomogram overestimates the probabilities [17].

Clinical value of the prediction models were estimated by decision curve analysis. It can compare net benefits of a prediction models with the scenes when all patients die or none. The x-axis and y-axis represent threshold probability and net benefit, respectively. Net benefit is calculated by benefits of the positives subtracting harms of the false positives [12]. If the prediction model has higher net benefits than the scenes when all patients die or none, it is considered of being clinical useful.

All analyses were conducted by SPSS 24.0 (Chicago, IL, USA) and the “survival”, “rms”, “rmda” and “suvivalROC” packages of R 3.6.1 (https://www.r-project.org/). *P*-value less than 0.05 was of statistical significance.

## Results

### Baseline characteristics

A total of 9876 cervical cancer patients were included. Baseline characteristics could be seen in Table 1. There were 2505 (25.36%) death over a median follow-up time of 42.43 (95% CI, 41.95 to 42.91) months. In all patients, the 3-year OS rate was 74.4%, and the 5-year OS rate was 67.7%. The survival curves were shown in Additional file 1: Fig. S1.

**Table 1:** Baseline characteristics, and results of univariate and multivariate Cox regression analysis

Variables	N (%)	Univariate analysis		Multivariate analysis	
		HR (95% CI)	*P-value*	HR (95% CI)	*P-value*
Age at diagnosis, years old					
< 45	4057 (41.079)	Reference		Reference	
45–59	3408 (34.508)	1.588 (1.436–1.756)	< 0.001	1.110 (1.001–1.231)	0.047
≥ 60	2411 (24.413)	2.748 (2.491–3.031)	< 0.001	1.536 (1.380–1.709)	< 0.001
Race					
White	7572 (76.671)	Reference		Reference	
Black	1204 (12.191)	1.679 (1.513–1.863)	< 0.001	1.282 (1.152–1.426)	< 0.001
Other	1100 (11.138)	1.062 (0.935–1.207)	0.356	1.083 (0.952–1.232)	0.226
Marital status at diagnosis					
Single	3020 (30.579)	Reference		Reference	
Married	4544 (46.011)	0.716 (0.652–0.787)	< 0.001	0.852 (0.773–0.940)	0.001
Other	2312 (23.410)	1.290 (1.169–1.424)	< 0.001	1.101 (0.992–1.223)	0.072
Grade					
I	1482 (15.006)	Reference		Reference	
II	4285 (43.388)	2.546 (2.121–3.055)	< 0.001	1.374 (1.138–1.659)	0.001
III	4109 (41.606)	4.690 (3.924–5.604)	< 0.001	1.732 (1.436–2.088)	< 0.001
Histology					
SCC	6363 (64.429)	Reference		Reference	
AC	3113 (31.521)	0.633 (0.576–0.696)	< 0.001	1.076 (0.974–1.189)	0.150
Other	400 (4.050)	2.585 (2.242–2.980)	< 0.001	1.757 (1.512–2.042)	< 0.001
Tumor size					
< 4 cm	5315 (53.817)	Reference		Reference	
≥ 4 cm	4561 (46.183)	5.084 (4.629–5.583)	< 0.001	1.635 (1.445–1.850)	< 0.001
FIGO stage					
INOS	39 (0.395)	Reference		Reference	
IANOS	94 (0.952)	0.432 (0.171–1.087)	0.075	0.543 (0.215–1.371)	0.196
IA1	1017 (10.297)	0.115 (0.054–0.243)	< 0.001	0.164 (0.077–0.350)	< 0.001
IA2	357 (3.615)	0.208 (0.094–0.464)	< 0.001	0.344 (0.154–0.768)	0.009
IB1	2774 (28.088)	0.320 (0.164–0.624)	0.001	0.547 (0.280–1.069)	0.078
IB2	713 (7.220)	0.869 (0.443–1.704)	0.682	0.912 (0.463–1.796)	0.789
IIA1	181 (1.833)	0.941 (0.454–1.950)	0.870	1.287 (0.620–2.671)	0.498
IIA2	261 (2.643)	1.515 (0.760–3.019)	0.238	1.369 (0.684–2.739)	0.375
IIB	885 (8.961)	1.203 (0.618–2.342)	0.586	1.207 (0.618–2.358)	0.581
IIINOS	25 (0.253)	2.503 (1.055–5.942)	0.037	2.625 (1.104–6.244)	0.029
IIIA	102 (1.033)	2.504 (1.224–5.123)	0.012	1.993 (0.971–4.093)	0.060
IIIB	2257 (22.853)	1.911 (0.991–3.686)	0.053	2.351 (1.208–4.574)	0.012
IVA	180 (1.823)	4.793 (2.437–9.426)	< 0.001	4.117 (2.082–8.141)	< 0.001
IVB	991 (10.034)	5.665 (2.934–10.936)	< 0.001	3.661 (1.473–9.104)	0.005
Cancer cells in lymph nodes					
Yes	2634 (26.671)	Reference		Reference	
No	7242 (73.329)	0.318 (0.294–0.344)	< 0.001	1.018 (0.912–1.137)	0.750
Metastasis					
Yes	1009 (10.217)	Reference		Reference	
No	8867 (89.783)	0.164 (0.150–0.179)	< 0.001	0.707 (0.378–1.320)	0.276
Primary site surgery					
Yes	6462 (65.431)	Reference		Reference	
No/Unknown	3414 (34.569)	4.729 (4.355–5.135)	< 0.001	1.476 (1.305–1.670)	< 0.001
Regional lymph node surgery					
Yes	4932 (49.939)	Reference		Reference	
No/Unknown	4944 (50.061)	3.401 (3.114–3.716)	< 0.001	1.691 (1.491–1.919)	< 0.001
Radiotherapy					
Yes	5792 (58.647)	Reference		Reference	
No/Unknown	4084 (41.353)	0.397 (0.362–0.536)	< 0.001	1.317 (1.171–1.482)	< 0.001
Chemotherapy					
Yes	5122 (51.863)	Reference		Reference	
No/Unknown	4754 (48.137)	0.390 (0.358–0.426)	< 0.001	1.714 (1.537–1.911)	< 0.001

### Independent prognostic factors

The results of univariate and multivariate Cox regression analysis could be seen in Table 1. Age at diagnosis, race, marital status at diagnosis, grade, histology, tumor size, FIGO stage, cancer cells in lymph nodes, metastasis, primary site surgery, regional lymph node surgery, radiotherapy and chemotherapy were the factors influencing on the overall survival of cervical cancer patients in univariate analysis. However, cancer cells in lymph nodes and metastasis were not statistically significant in multivariate analysis. Therefore, eleven variables were the independent prognostic factors of the nomogram.

### Development and internal validation

**Fig. 1:**
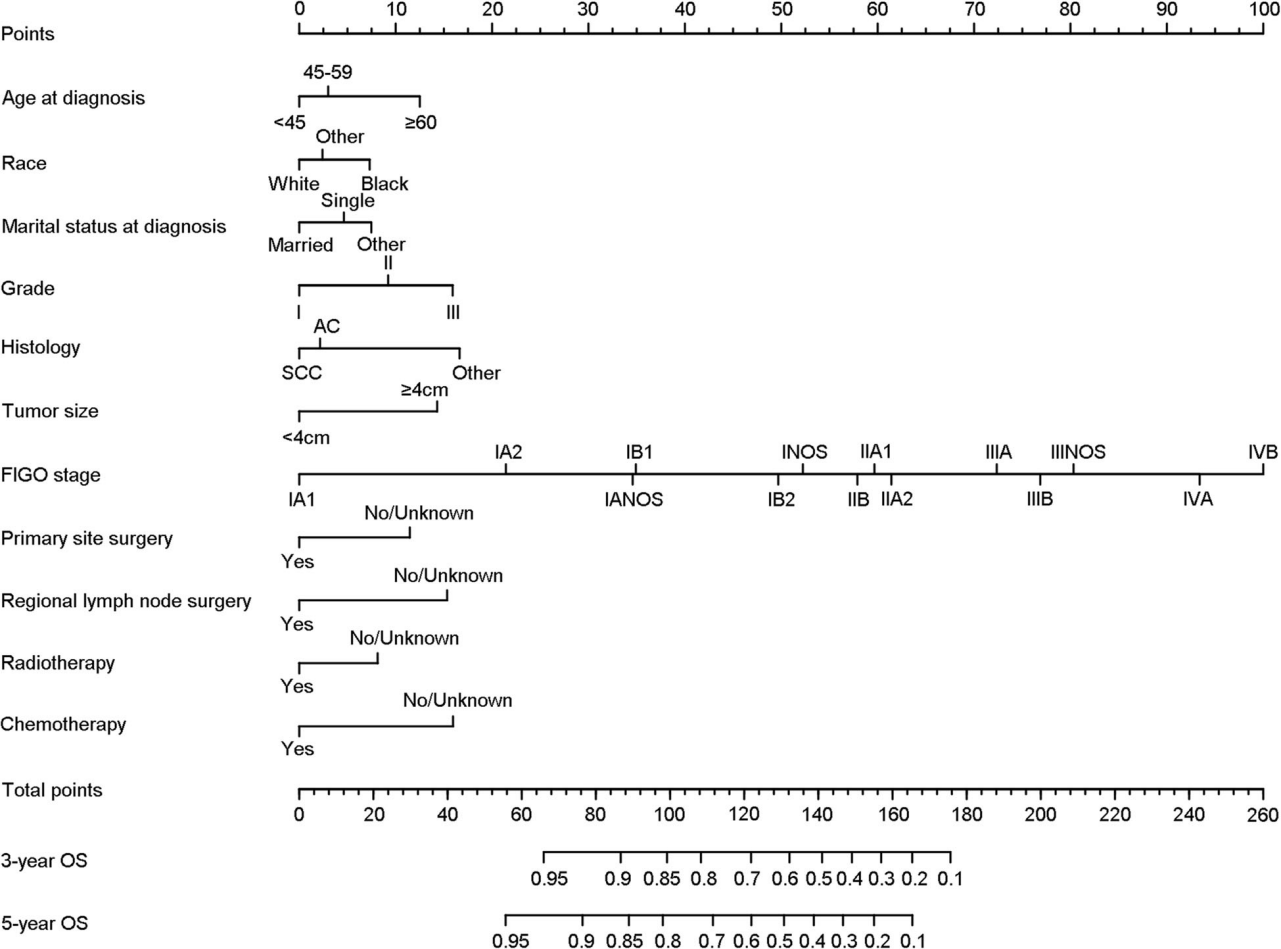
Nomogram for predicting the overall survival probability in patients with cervical cancer

**Fig. 2:**
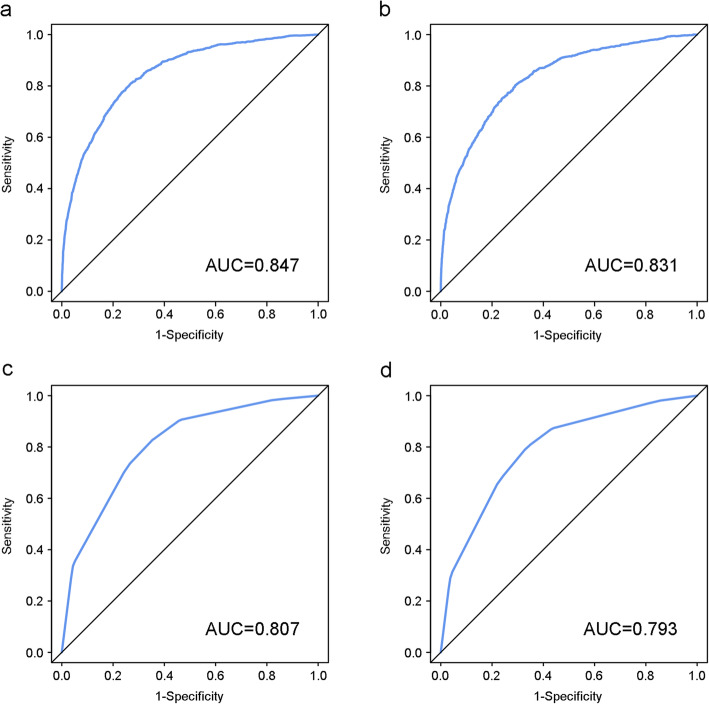
ROC curves for 3-year and 5-year OS of the nomogram and FIGO stage prediction model. (a) ROC curve for 3-year OS of the nomogram, (b) ROC curve for 5-year OS of the nomogram, (c) ROC curve for 3-year OS of the FIGO stage prediction model and (d) ROC curve for 5-year OS of the FIGO stage prediction model

**Fig. 3:**
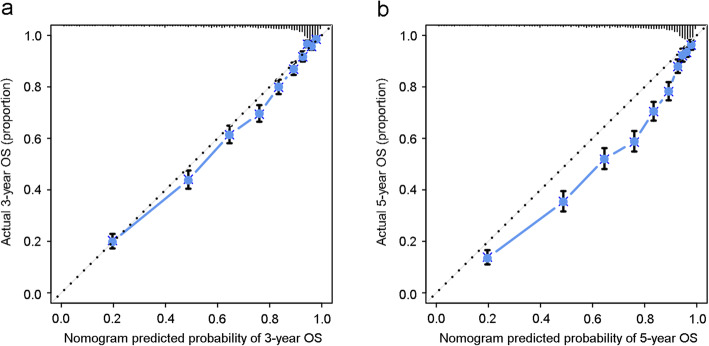
Calibration plots for (a) 3-year and (b) 5-year OS of the nomogram

The independent prognostic factors were entered to develop the nomogram (Fig. 1). In the nomogram, FIGO stage shared the largest contribution, followed by histology, grade and chemotherapy. The sociodemographic factors (age at diagnosis, race and marital status at diagnosis) also partially dedicated to the nomogram. The C-index of the nomogram was 0.826 (95% CI: 0.818 to 0.834), which was better than that of the FIGO stage prediction model (C-index: 0.785, 95% CI: 0.776 to 0.793). ROC curves for the nomogram and FIGO stage prediction model were shown in Fig. 2. AUC of the nomogram in 3-year and 5-year OS prediction were 0.847 and 0.831 respectively, which were higher than those of the FIGO stage prediction model (0.807 in 3-year OS prediction and 0.793 in 5-year OS prediction). The calibration plots of nomogram were shown in Fig. 3. The nomogram had good calibration when predicting 3-year OS probability. But when it came to 5-year OS prediction, it had poor calibration and was overfitting and overestimated the probabilities.

### Clinical usefulness

**Fig. 4:**
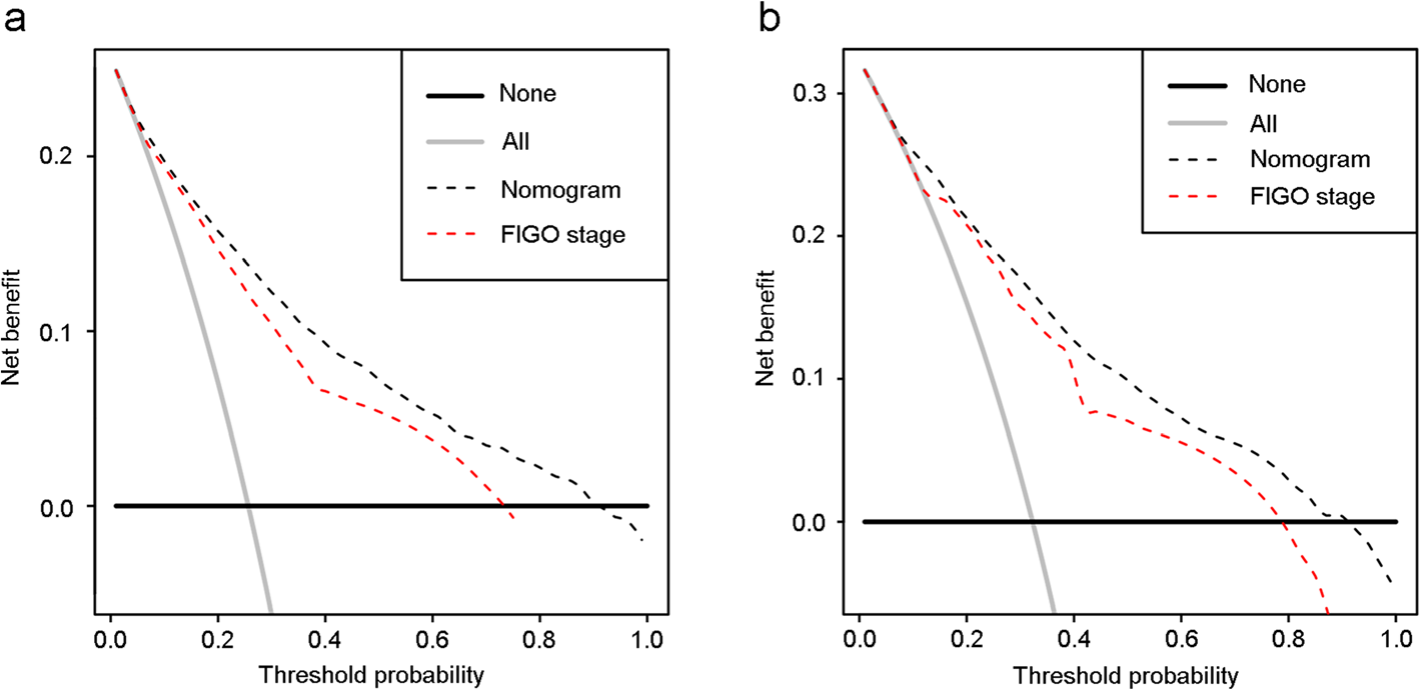
Decision curves of the nomogram and FIGO stage prediction model. (a) Decision curves for 3-year OS prediction, and (b) decision curves for 5-year OS prediction

Decision curves for the nomogram and FIGO stage prediction model were showed in Fig. 4. In 3-year OS prediction, when the threshold probability was between 0 and 90%, the net benefits of the nomogram were better than the scenes when all patients died or none. In 5-year OS prediction, when the threshold probability was between 4 and 91%, the net benefits of the nomogram were better than the scenes when all patients died or none. And the net benefits of the nomogram were higher than those of the FIGO stage prediction model.

## Discussion

A nomogram was developed to calculate 3-year and 5-year OS probability in patients with cervical cancer. The nomogram comprised eleven predictors on the sociodemographic characteristics and clinical treatment information. The discrimination of the nomogram was better than the FIGO stage prediction model. The calibration of the nomogram was great in 3-year OS prediction, but poor in 5-year OS prediction. In addition, the nomogram had higher net benefit than the FIGO stage prediction model.

We found that FIGO stage was not the only factor influencing the prognosis of cervical cancer, and sociodemographic characteristics and clinical treatment information were also important. For married women, they have sex life, which is an important factor of the occurrence of cervical cancer. But they could also get external support from their husband, which is a protective factor [18]. The influence of histology on the prognosis of cervical cancer has long been debated [19, 20]. A study reported that adenocarcinoma had negative relation with the survival of advanced stage cervical cancer [21]. Tough FIGO stage system was widely used in clinical activities, the survival time of cervical cancer was diverse even with identical stage. Therefore, the FIGO stage is not satisfactory enough when used to predicted prognosis. The nomogram could reduce the diversity due to different treatment and sociodemographic status when predicting prognosis of cervical cancer. And we found that the nomogram performed better than the FIGO stage prediction model on precise prognosis.

Nomogram is widely used as diagnosis device to predict the probability of patients suffering from diseases and the prognosis of malignance [22–25]. But for the prognosis of cervical cancer, only a few studies applied it to visualize prediction models [10, 11]. In previous studies, most were based on small sample size cohorts, which might reduce the robustness and generalizability of the nomograms [10, 26]. Our nomogram was based on a cohort with large sample size, and it guaranteed the reliability and generality of the result. Some researchers developed nomograms to predict survival of cervical cancer with certain FIGO stages or specific treatments [27–29]. Marchetti et al. [27] estimated the survival of stage IB2-IIIB cervical cancer after curative chemotherapy and radical surgery with nomogram. The C-indexes of the previous nomograms tended to be between 0.65 and 0.75, which were acceptable [10, 23, 30]. The C-index of our nomogram was 0.826 (95% CI, 0.818 to 0.834), indicating that the discrimination ability of our monogram was excellent. In addition, the C-index of the nomogram was better than that of FIGO stage prediction model. For current reports with nomogram, most are with great calibration, and their calibration plots are close to the ideal line [10, 23, 30]. In our study, the nomogram had good calibration in 3-year OS prediction. However, the calibration plot of the nomogram deviated from the ideal line in 5-year OS prediction. It might be owing to the inappropriate proportion of censored events. In this study, there were too many censored events covering over 70% of total patients. The increased censorship might lead to decreased accuracy and effectiveness, and increased bias of the prediction model [31]. Despite that, this nomogram was still meaningful as its calibration was good in 3-year OS prediction.

Decision curve analysis puts benefit and harm together to measure net benefit of diagnosis method or prediction model [12]. Compared with traditional ROC curve, decision curve analysis is better, because it takes clinical usefulness into consideration. Clinical usefulness is an important judging indicator whether a prediction model can be truly used in clinical activities and patients can benefit from it. As far as we know, the number of papers applying this new method to assess the net benefit of prediction models is very small. In some high-quality papers, researchers used it to assess the clinical usefulness of their prediction models about venous thromboembolism and gestational diabetes mellitus [24, 25]. But there are only a few papers applying it to prediction models for cervical cancer survival. Zhang et al. [32] measured the net benefit of their risk assessment system for estimating survival time of distantly metastatic cervical cancer, and found that it was of clinical utility. In this study, we not only calculated the net benefit of the nomogram but also the FIGO stage prediction model. And we found that the net benefit of the nomogram was higher than the FIGO stage prediction model, indicating that our nomogram was clinical useful.

For patients with cervical cancer, the key point they are concerned about might be that how long they will live. Tough the FIGO stage system is currently available in clinical activities, the survival time of cervical cancer has a wide spectrum even with identical FIGO stage. Our study successfully developed a nomogram to predict the survivorship of cervical cancer patients, which was composed of sociodemographic and clinical treatment information. It could provide more comprehensive and more accurate prognostic prediction than the traditional FIGO stage system and it could be proposed as a complement of the FIGO stage system. The nomogram was simple-to-use and it could be used as a paper-based or online prediction tool to predict prognosis of cervical cancer before and after treatment, so that it could help clinicians make the optimal strategic decisions, provide individualized clinical care and consultation to meet the needs of patients. Moreover, it could aid clinicians to distinguish patients who might have more benefits from treatments, carry out clinical trials and make tailored follow-up plans. In short, the nomogram was helpful both in clinical treatment and research.

However, our study still had some limitations. Firstly, the nomogram did not contain some factors related to cervical cancer, such as lymph vascular space invasion. Umezu et al. [33] found that lymph-vascular space invasion was one of the prognostic factors of the overall survival of cervical cancer patients who were staged IA-IIA and underwent surgical resection. And Srisomboon et al. [34] identified that lymph vascular space invasion was an important factor influencing the survival of cervical cancer. Because information of lymph-vascular space invasion for cervical cancer patients was blank in the SEER database, we did not include this significant factor in this study. Besides, the SEER database did not have details of the chemotherapy, such as the use of targeted drug, which was critical for the prognosis of cervical cancer. And it lacked the information of living surroundings, lifestyle, adjuvant therapy and commodities, so we could not get all prognostic factors into consideration, which was an intrinsic limitation of SEER-based study. However, this nomogram embodied acceptable performance with present prognostic factors. Secondly, different treatments might have different impacts on the prognosis of cervical cancer. We failed to subdivide each treatment and only divided them by whether it was performed or not. For primary site surgery, there are many surgery methods for cervical cancer, such as conization of uterine cervix, total hysterectomy removes, et al. Patients with different surgery methods might have different outcome. Thirdly, we did not conduct external validation to further assess this nomogram.

## Conclusions

In conclusion, we developed a clinical useful nomogram for calculating overall survival in cervical cancer patients, based on sociodemographic and clinical related information. It performed better than the FIGO stage prediction model and could help clinicians to choose optimal treatments and precisely predict prognosis in clinical care and research.

## Supplementary information

**Additional file 1: Fig. S1.** Kaplan-Meier OS curves for patients with cervical cancer. Each Kaplan-Meier OS curve was stratified by (a) all, (b) age at diagnosis, (c) race, (d) marital status at diagnosis, (e) grade, (f) histology, (g) tumor size, (h) FIGO stage, (i) cancer cells in lymph nodes, (j) metastasis, (k) primary site surgery, (l) regional lymph node surgery, (m) radiotherapy and (n) chemotherapy, respectively.

## Data Availability

The data of this study are available from the Surveillance, Epidemiology, and End Results (SEER) database (https://seer.cancer.gov/).

## Abbreviations

OS: Overall Survival
SEER: Surveillance, Epidemiology, and End Results
SCC: Squamous Cell Carcinoma
AC: Adenocarcinoma
FIGO: International Federation of Gynecologists and Obstetricians
HR: Hazard Ratio
CI: Confidence Interval
