# Supplementary figures and images for: Calculating the overall survival probability in patients with cervical cancer: a nomogram and decision curve analysis-based study
Source: BMC Cancer. 2020 Sep 1;20:833. doi: 10.1186/s12885-020-07349-4 (PMC7466454; doi:10.1186/s12885-020-07349-4)

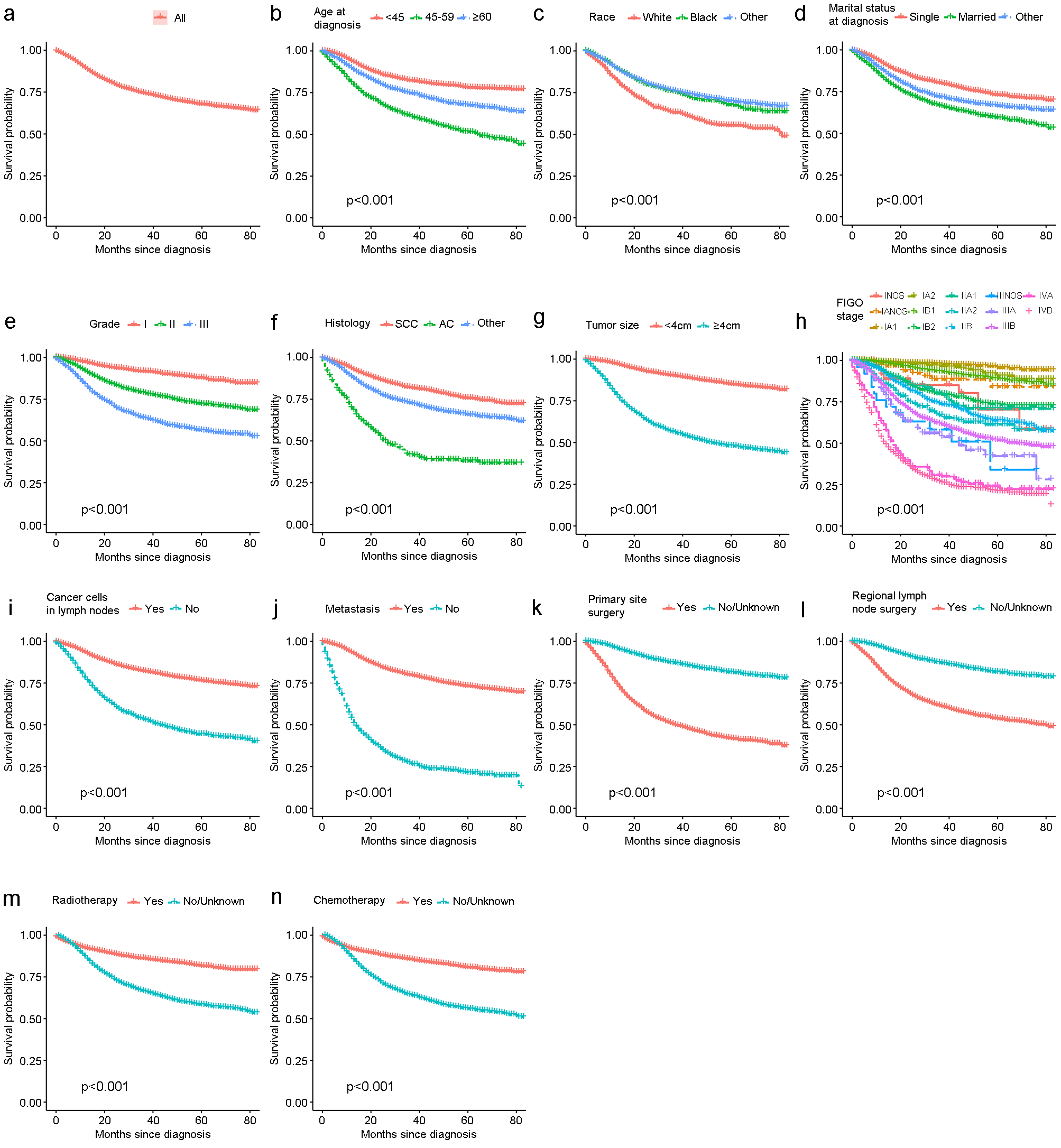

Supplement: Supplementary file 1 — Additional file 1: Fig. S1. Kaplan-Meier OS curves for patients with cervical cancer. Each Kaplan-Meier OS curve was stratified by (a) all, (b) age at diagnosis, (c) race, (d) marital status at diagnosis, (e) grade, (f) histology, (g) tumor size, (h) FIGO stage, (i) cancer cells in lymph nodes, (j) metastasis, (k) primary site surgery, (l) regional lymph node surgery, (m) radiotherapy and (n) chemotherapy, respectively. [file 12885_2020_7349_MOESM1_ESM.tif]
